# Are children with tuberculosis in Pakistan managed according to National programme policy guidelines? A study from 3 districts in Punjab

**DOI:** 10.1186/1756-0500-3-324

**Published:** 2010-11-30

**Authors:** Nauman Safdar, Sven Gudmund Hinderaker, Noor Ahmed Baloch, Donald A Enarson, Muhammad Amir Khan, Odd Morkve

**Affiliations:** 1 Association for Social Development, Islamabad, Pakistan; 2 Centre for International Health, University of Bergen, Norway; 3 National TB Control Programme Pakistan; 4 International Union against Tuberculosis and Lung Disease, Paris, France

## Abstract

**Background:**

The adherence to policies of National TB Control Programme (NTP) to manage a case of tuberculosis (TB) is a fundamental step to have a successful programme in any country. Childhood TB services faces an unmet challenge of case management due to difficulty with diagnosis and relatively new policies. For control of childhood TB in Pakistan, NTP developed and piloted its guidelines in 2006-2007. The objective of this study was to compare the documented case management practices of pediatricians and its impact on the outcome before and after introducing NTP policy guidelines.

**Findings:**

An audit of case management practices of a historical cohort study was done in children below 15 years who were put on anti-tuberculosis treatment at all nine public hospitals in three districts in province of Punjab. The study period was two years pre-intervention (2004-05) and two years post-intervention (2006-07) after implementation of new NTP policy guidelines for childhood TB. There were 920 childhood TB cases registered during four years, 189 in pre-intervention period and 731 in post-intervention period. The practices changed significantly in post-intervention period for use of tuberculin skin test (63% of pulmonary cases, 19% of extrapulmonary cases and 67% for site unknown), and for the use of chest x-ray (69% of pulmonary cases, 16% of extrapulmonary cases and 74% for site unknown). Diagnostic scores were recorded for only a minority of cases (18%). The proportion of correct drugs pre- and post-intervention remained same. There were unknown treatment outcomes in 38 out of 141 cases (27%) in pre-intervention and in 483 out of 551 cases (87%) post-intervention, all among the 692 cases without documented treatment supporter.

**Conclusions:**

The study has shown that pediatricians have started following parts of the national policy guidelines for management of childhood TB. The documented use of diagnostic tools is increased but record keeping of case management practices remained inadequate. This seems to increase case finding substantially but the treatment outcomes were poor mainly due to unknown outcomes. Development and implementation of standardized operational tools and regular monitoring system may improve the services.

## Background

The adherence to policies of the National TB Control Programme (NTP) to manage a case of tuberculosis (TB) is a fundamental step to a successful programme in any country. Since the initiation of the campaign to control tuberculosis, a positive change has been observed in managing TB cases in the Eastern Mediterranean region [1] with e.g Pakistan reaching 100% DOTS coverage in year 2005 meaning all districts having a TB control setup. Currently the NTPs in the high TB burden countries are dealing with the components of the TB control strategy within which management of childhood TB remains an essential component for those to be offered care [2].

The diagnosis of pediatric tuberculosis is usually made on the basis of clinical evidence and is difficult particularly in infants and young children in whom the symptoms are often non-specific [3]. Several studies have documented the poor diagnosis and treatment practices of clinicians while managing a case of tuberculosis [4-6]. The World Health Organization (WHO) has published guidelines on the diagnosis and management of TB in children in 2006 [7]. There are several diagnostic criteria that have been detailed in the guidelines including the use of clinical symptoms, sputum microscopy, chest radiograph (CXR), tuberculin skin test (TST) and scoring charts. Childhood TB services face a challenge of case management due to difficulty with diagnosis in clinical practice [8] and the recent introduction of international policies [7].

For the control of childhood TB in Pakistan the NTP has developed and piloted its guidelines in 2006-2007 in the public sector hospitals in ten selected districts. The policy guidelines were developed in collaboration with the Pakistan Pediatric Association and consist of protocols with a purpose to improve care of children with TB [9]. The guidelines were introduced by training pediatricians in the pilot districts, supplying anti-TB drugs, introduction of TST and monitoring support. A retrospective review of the piloting experience in three of these districts was conducted to compare the case notification and treatment outcomes of children with TB in 2004-5 with 2006-7 [10]. The objective of this study was to compare the documented case management practices of pediatricians and its impact on the outcome before and after introducing the NTP policy guidelines. Our hypothesis was that the implementation of new NTP policy guidelines would improve the case management practices and the treatment outcomes among children with tuberculosis.

## Method

### Study design

An audit of the case management practices in a historical cohort was done by reviewing patient records. We included all children aged less than 15 years who were registered as cases of TB at the nine public hospitals (district and sub-district) in three districts of the province of Punjab. Children detected with TB at health facilities other than these hospitals such as maternal, child and primary health facilities, private clinics and para-statal hospitals were not included in the study. The study received ethical clearance from the National Bioethics Committee of Pakistan.

### Diagnosis and treatment criteria

**Figure 1:**
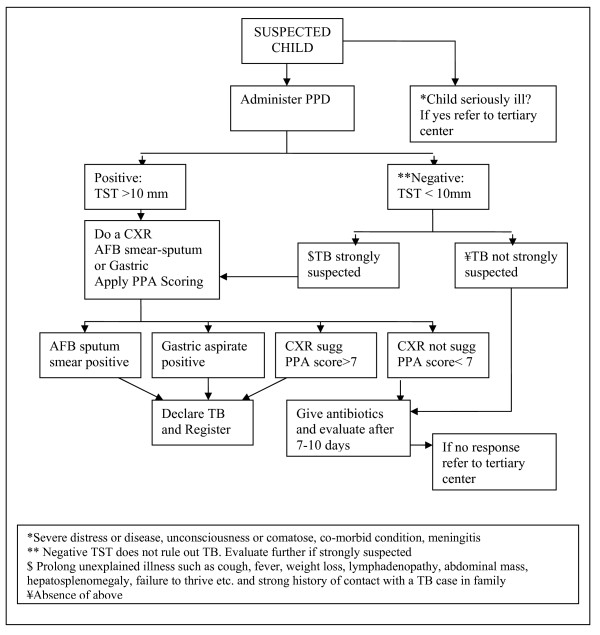
**NTP flow chart for evaluation of a child with suspected TB. *Source: Ref ***[9]

The diagnosis was based on the combination of history and examination suggestive of TB, history of close contact with an adult case of TB, bacteriological examination, chest X-ray, TST and histopathology of tissue samples. In addition there was a scoring chart meant to help in making diagnostic decisions. A diagnostic algorithm has been presented by NTP in the guidelines to facilitate the case management process (Figure 1). These criteria have been part of WHO guidelines [7] and NTP Pakistan policy guidelines [9] related to management of children with TB. The prescription protocols were also part of the policy guidelines. The prescription was based on body weight of the patient and the category of disease. The four treatment categories were as follow: Category-I was pulmonary TB, severe forms of new extra-pulmonary TB, new severe concomitant HIV disease and TB meningitis. Category-II was previously treated smear positive pulmonary TB, relapse, treatment after interruption and treatment failure. Category-III was new smear negative pulmonary and less severe forms of extra-pulmonary TB and Category-IV was chronic and multi-drug resistant (MDR) TB. The drugs prescribed to a child diagnosed with category I-III TB always have an intensive phase of 2 to 3 months duration and a continuation phase of 4 to 6 months duration. The anti-TB drug dosage depends on body weight and category of patient. Category I, is 2 months of Isoniazid (H) plus Rifampicin (R) plus Pyrazinamide (Z) plus Ethambutol (E) followed by 4 months of H and R (abbreviated 2HRZE/4RH), for category II, it is 2HRZES/1HRZE/5HRE, for category III, it is 2HRZ/4HR and for category IV, treatment is individualized for each patient.

### Childhood TB care services

The district level public sector hospitals in Pakistan usually have 'pediatrician', i.e. child specialist or doctors and health personnel taking care of children. They were informed by NTP on how to manage childhood TB including suspect screening, use of scoring chart, diagnosis and managing records. Facilities that are available in these hospitals include sputum smear microscopy and CXR to support TB diagnosis. The administration of TST is delegated to a nurse or vaccinator. The recording and reporting (R&R) of routine TB cases is the responsibility of a DOTS facilitator, usually a paramedic from the existing set up and trained on NTP routine adult TB R&R system.

The term 'practice' in this study is the pediatrician's documented record of case management which should be based on NTP childhood TB policy guidelines. The absence of such records was termed as 'missing information'. The *missing *in terms of diagnosis includes missing information on the use of TST, chest X-ray or score charts. In the case of prescribing anti-TB treatment *inadequacy *was used to denote prescriptions that were wrong according to category and dose of any single drug.

### Study sites

The NTP selected ten districts in all four provinces of Pakistan to pilot their childhood TB policy guidelines. The selection of districts was based primarily on evidence of functioning adult TB care in a district, geographic distribution and access, and willingness of districts to participate in a pilot. For our retrospective study, we selected all three districts from the province of Punjab for the study because of continued programme support including drugs and materials, which facilitated implementation under programme circumstances. All nine hospitals at district and sub-district level were included. The district level hospitals have more specialist persons and inpatient facilities available as compared to sub-district level hospitals. Otherwise they are almost similar in the context of routine TB care services including childhood TB case management. These three study districts represent southern, central and northern parts of the province. All childhood TB cases registered at these hospitals during the period under review were included in the study [11]. The pediatricians from these hospitals were oriented by NTP on the newly developed national policy guidelines. The tablet formulation of childhood anti-TB drugs was provided along with the supply of TST and required print materials. The X-ray service was available as a part of regular hospital arrangement.

### Data collection and analysis

The documented case management practices in two cohorts of childhood TB cases were compared. The first cohort included patients registered during the two complete years 2004 and 2005, when NTP had no particular emphasis on childhood TB ('pre-intervention') and had recently expanded the DOTS strategy for adults. The second cohort consisted of patients registered during 2006 and 2007, when new NTP childhood TB policy guidelines were being implemented ('post-intervention'). The prime sources of data were the TB registers, TB treatment cards and quarterly reports. These were mainly available as outpatient records and were part of the standard NTP recording and reporting system. A specially designed form was provided in year 2006 to the pediatricians to document the scores of individual children using the scoring chart. The TB patient registration and quarterly reporting in the public sector had been decentralized in Pakistan to the level of diagnostic centers including all the public hospitals and the rural health centers in the district. These diagnostic centers (average 12-15 in public sector in a district) register the TB cases including children and send their quarterly case finding and treatment outcome reports to the district TB coordinator office. The district TB coordinator office then consolidates these quarterly reports and transmits a quarterly consolidated district case finding and treatment outcome report to the provincial TB control office. There are regular quarterly intra- and inter-district meetings to consolidate reports to avoid errors and to facilitate the progress. A researcher visited each hospital to review the patient records and extract relevant data, using a specially designed manual tool. The quality of data extraction was ensured by another researcher by cross-checking the extracted data for missing information and inconsistency.

Data were entered and analyzed using the SPSS version 15 software package. The comparisons of the two cohorts relate to diagnostic practices, to prescription of anti-TB drugs, allocation of treatment support and to treatment outcomes. For comparing group differences of categorical variables, Pearson Chi-square test was used and Student t-test was used for continuous variables. The level of significance was set at p < 0.05.

## Results

### Criteria used for case management

There were 920 childhood TB cases registered during the four years in the three districts studied, 189 in the pre-intervention period and 731 in the post-intervention period (table 1). The age distribution of children was as follows: 93 (10%) below 2 years of age, 202 (22%) between 2-5 years and 608 (66%) between 6-14 years and in 17 (2%) the record on age was missing.

**Table 1:** Diagnostic practices documented by site of TB during two periods in public health facilities in three districts of Pakistan

	Pre-intervention (2004-05) *n(%)*				Post-intervention (2006-07) *n(%)*			
	0-14 years		0-5 y	6-14 y	0-14 years		0-5 y	6-14 y
**Age groups (years)**	189				731			
**Pulmonary TB**	109	(100)			501	(100)		
TST	0		0	0	316	(63)	195	119
X-ray chest	16	(15)	0	16	346	(69)	195	149
Sputum smear	94	(86)	1	93	159	(32)	3	156
Scores available	0		0	0	75	(15)	42	33
Biopsy	0		0	0	0		0	0
**Extra-pulmonary TB**	75	(100)			127	(100)		
TST	0		0	0	24	(19)	14	10
X-ray chest	2	(3)	0	2	20	(16)	9	11
Sputum smear	8	(11)	1	7	1	(1)	0	1
Scores available	0		0	0	9	(7)	6	3
Biopsy	1	(1)	1	0	3	(2)	0	3
**Site unknown**	5	(100)			103	(100)		
TST	0		0	0	69	(67)	41	28
X-ray chest	0		0	0	76	(74)	44	32
Sputum smear	0		0	0	5	(5)	0	5
Scores available	0		0	0	47	(46)	31	16
Biopsy	0		0	0	0		0	0

Table 1 presents a comparison of diagnostic practices in the two periods. The proportion of cases in which no information was available to assess whether the case was pulmonary or extrapulmonary was relatively low in the pre-intervention period (5 cases, 2.6%) but was significantly higher in the post-intervention period (103 cases, 14.1%). In the pre-intervention period, none of the patients had a tuberculin skin test or a diagnostic score recorded. Sputum smear was the most frequently used diagnostic tool in the pre-intervention period for both pulmonary and extrapulmonary cases. A significant change occurred in the post-intervention period for the TST (63% of pulmonary, 19% of extrapulmonary cases and 67% for site unknown), and for the chest x-ray (69% of pulmonary, 16% of extrapulmonary cases and 74% for site unknown). Diagnostic scores were recorded for only a minority of cases (15% of pulmonary, 7% of extrapulmonary cases and 46% for site unknown).

### Treatment practices

Out of all 920 cases registered during 2004-07 only 343 (37%) had a recorded patient category and these were correct in 96%. Prescription of correct drug regimen increased in absolute numbers from 46 pre-intervention to 167 post-intervention after the implementation of guidelines. However the proportion of correct drugs regimen pre- and post-intervention remained the same, 24% and 23%, respectively.

### Association with treatment outcome

During the study the treatment support allocation was recorded among 228 (25%) of all the registered cases (table 2). The proportion with recorded treatment supporter remained unchanged from pre-intervention period 48 (25%) to post-intervention period 180 (25%). Most cases were allocated to health care workers offering treatment support (188 cases, 21%) as compared with family member treatment support (40 cases, 4%). Out of the total 920 cases notified during four years 692 (75%) were found in the group without treatment support; 141/189 (75%) in pre-intervention period and 551/731 (76%) in post-intervention period. There were unknown treatment outcomes in 38 out of 141 cases (27%) pre-intervention and in 483 out of 551 cases (87%) post-intervention, and were among the 692 cases with no documented treatment supporter. Among cases with treatment supporter unknown treatment outcomes were 3 (6%) in pre-intervention and 64 (36%) post-intervention.

**Table 2:** Treatment outcomes by treatment supporter assigned, among the same cases (n = 920) as in table 1

**Treatment Outcome**	**With treatment supporter**					**Without treatment supporter**				
	
	**2004-05 *n(%)***		**2006-07 *n(%)***		***P *value**	**2004-05 *n(%)***		**2006-07 *n(%)***		***P *value**
	
Cured	16	(34)	11	(6)	<0.05	8	(6)	12	(2)	<0.05
Treatment completed	24	(50)	88	(49)	0.9	62	(43)	48	(10)	<0.05
Died	1	(2)	0		0.05	1	(1)	0		<0.05
Default	3	(6)	15	(8)	0.6	29	(21)	7	(1)	<0.05
Transfer out	1	(2)	2	(1)	0.6	3	(2)	1	(0.2)	<0.05
Unknown	3	(6)	64	(36)	<0.05	38	(27)	483	(87)	<0.05
Total	48	(100)	180	(100)		141	(100)	551	(100)	

## Discussion

In our study we have shown that there was a substantial increase in the childhood TB case notification from 189 pre-intervention to 731 post-intervention after the implementation of new policy guidelines. The NTP provided the training on policy guidelines for childhood TB and logistic support including TST and anti-TB drugs to initiate the childhood TB care in the public sector district level hospitals. Before these NTP policy guidelines adult anti-TB drugs were provided free of cost and the individual dosage was given per kg bodyweight. Later the child specific anti-TB drugs were also provided along with TST. Based on available information we cannot say how much of the increase in TB notification in children is due to guidelines or logistic support or other issues. The training was provided to the pediatrician and not to the DOTS facilitator (paramedics and nurses) who were supposed to be involved in the R&R activity. This resulted in weak implementation of R&R with incompleteness of records. The use of tuberculin skin test after introduction of the NTP guidelines increased. The TST was not initially available from the programme and after the implementation of the new policy its use by the pediatricians in the programme conditions was encouraging. The TST as a tool to support diagnosis of TB among children is well established and still considered to be the best available method to detect TB infection [12]. It can be used as an adjunct in diagnosing TB disease [13] and a regular stock of tuberculin should be maintained in hospitals involved with managing childhood TB [14].

The documented use of chest radiographs to assist in diagnosis increased among both pulmonary and extra-pulmonary TB cases. The use of CXR in making the diagnosis of children with TB has been supported internationally and a majority of cases with children with pulmonary TB have CXR changes suggestive of TB [13]. The CXR should always be read by a trained health care provider. Since NTP adopted the DOTS strategy in 1995 the emphasis remained on the use of sputum smear microscopy which downplayed the importance of CXR in supporting the diagnosis of smear negative TB. In a study in Malawi over 80% of the childhood TB cases the CXR finding were consistent with TB [4]. The increased use of CXR emphasizes the need of structured training of heath care providers on reading the CXR among children with TB; if not done it may have consequences on the quality of diagnosis and care. The NTP policy guidelines had section on the use of X-ray to help the pediatrician diagnose a child with suspected TB. The quality control mechanism was not well defined with a possibility of missed cases or over diagnoses. To address this limitation the NTP Pakistan has recently developed a training module having sections on reading chest X-ray in children, adapted from international literature, which will address this important issue related to case management. There are also international guidelines available to assist the health care workers in low income countries to interpret chest radiographs of children suspected of having TB [15]. The diagnosis of TB in low-income countries needs to be improved based on the available technology and resources [14].

**Figure 2:**
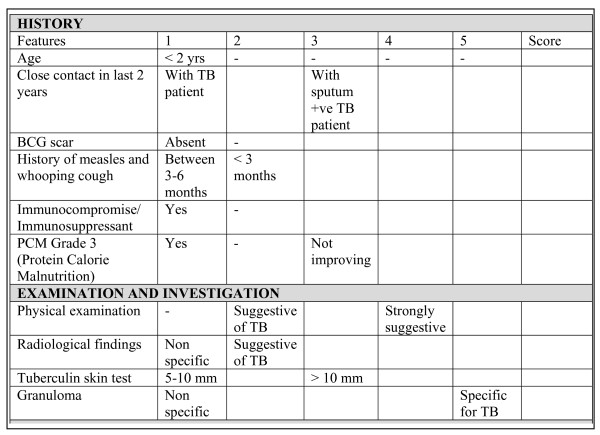
**NTP and Pakistan Pediatric Association scoring chart for screening of TB in children. *Source: Ref ***[9].

In our study some children had sputum smear done, although this did not increase with introduction of the new policy guidelines. Very few of the cases below 5 years of age got sputum smear done which shows the challenge faced by the health care providers in getting smear done in very young children within routine programme conditions. The NTP childhood TB case management policy and involving pediatricians in TB control activities were recent activities. We observed an increase in case notification during post-intervention phase but the linkage between various investigation facilities to operationalize diagnostic algorithms within the hospital was limited. The role of laboratory services in childhood TB diagnosis including sputum smear microscopy and specimen collection by gastric lavage, was not well defined. The operational linkages between the pediatricians and laboratory services need to be strengthened in order to enhance sputum microcopy among children suspected with TB. Similar operational issues are relatively common in the start of a new initiative and gradually improve with programme implementation. More training is required for introducing techniques such as gastric lavage and induced sputum within routine programme situation in order to increase sputum smear microscopy among children. The use of the score chart was introduced by the new guidelines. The score chart helps suspect screening [7] and the NTP policy-aim is to have a score of each case diagnosed. The use of a score chart has been suggested in the situations where accurate diagnosis of childhood TB is complicated by the lack of diagnostic tools and facilities [16]. The diagnostic criteria documented in the score chart are simple and well structured (Figure 2) and can be applied in a resource poor setting like the health facilities in Pakistan [17]. More attention needs to be paid by the health care workers to document the use of score charts.

The study has shown that the proportion of cases with correct regimen has not changed from pre-intervention to post-intervention. There is wide variation in the practices of pediatricians regarding prescribing anti-TB drugs to the diagnosed children and none of the doses prescribed were found to be recorded correctly. To have good treatment outcomes the application of standardized treatment regimen needs to be according to the relevant diagnostic category [18]. This suggests that NTP could benefit from more stringent process monitoring of the childhood TB care to improve practices. The treatment support and treatment outcome were recorded in a low proportion of the children and the proportion of cases documented as cured even lower in the post-intervention, than in the pre-intervention period. The purpose of our study was to compare practices before and after introduction of guidelines, and not the difference between districts or hospitals; enhanced childhood TB case finding has shown variations across the three districts studied, which may related to difference of individual practices. We have shown in a previous paper that the high number of cases notified and the high number of unknown outcome (pre-intervention 21.7%, post-intervention 73.3%) were from one of the three districts because of absence of adequate measures to case follow-up [10]. In studies conducted in Malawi and Papua New Guinea unknown outcome was reported in 21% and 23% of notified cases [19,20]. The training and logistic inputs remained standard for all these districts in the post-intervention period. The incomplete reporting of treatment outcomes may be due to the practitioner's level of commitment, work load and limited experience with the new intervention. Similarly the patient/families may have failed to adhere to treatment due to socio-economic reasons. These issues are more common at the start of the programme and gradually settle down with more structured monitoring of process of care and outputs, involving the community volunteers in treatment support, awareness and social mobilization.

Patient treatment cards are recommended for documenting treatment adherence [18] and children with TB should always be included in the routine NTP recording and reporting system [21]. It is an advantage for the childhood TB control that adequate attention should be given on the R&R system. Pediatricians should be engaged in diagnosis and treatment. The already trained and available personnel should be oriented and involved in R&R to capture childhood TB cases, with a provision of additional human resource where required, so that all clinicians dealing with childhood TB are trained to do it. This further supports the arguments that with systematic improvement of childhood TB case management practices in the public sector we could achieve good results.

A strength of this retrospective study is that it is based on the review of available patient records under routine programme conditions at nine selected hospitals in public sector in three districts with previous adult TB care experience and good collaboration with NTP. One major limitation is that many patient records were incomplete and it was not possible to get more information on each of the patients from the hospitals.

## Conclusions

The study has shown that pediatricians have started following parts of the national policy guidelines for management of childhood TB. In the diagnosis of childhood TB the documented use of TST, CXR and score chart increased but the same was not observed for sputum smear which actually declined. Record keeping of case management practices including diagnosis and treatment support remains inadequate. The impact of these practices on treatment outcomes was scanty with too many unknown outcomes. Perhaps the absence of operational tools to guide the process of care and monitoring to improve the case management practices and outcomes confines the achievement of desired results. Development and implementation of standardized operational tools and regular monitoring system is required to improve the performance and quality of services.

## Competing interests

The authors declare that they have no competing interests.

## Authors' contributions

NS has participated in study design, data entry and analysis, data interpretation, draft writing, editing and submission. SGH has participated in study design, data analysis, and data interpretation and editing whereas NAB, DAE, MAK and OM have participated in study design and data interpretation and editing. All authors read and approved the final manuscript.
